# Contributors to and Consequences of Poor Sleep in Older Adults: Biopsychosocial Perspectives

**DOI:** 10.1093/geroni/igaa057.1923

**Published:** 2020-12-16

**Authors:** Adam Spira, Katie Stone

## Abstract

Sleep is a significant contributor to health and wellbeing across the lifespan, especially in later life. Poor sleep is common among older adults and can be both a risk factor for and consequence of numerous physical and mental health-related outcomes. In this symposium, we will present novel results from four studies that will advance understanding of the biological, psychological, and social factors that may contribute to or result from poor sleep in older adults. Specifically, Study 1 will present findings tying objectively measured sleep to performance on cognitive tasks administered using ecological momentary assessment (EMA) in the day-to-day lives of older adults with or without mild cognitive impairment (MCI). Study 2 will examine associations of personality dimensions and facets with insomnia symptoms in well-functioning older adults. Study 3 will examine psychological pathways linking parent-child relationships to subjective and objective sleep characteristics among older parents. Finally, study 4 will examine use patterns of cannabis for the treatment of sleep problems in older adults, and the ways in which this might differ from patients using cannabis for other reasons (e.g., pain). Together, this symposium will highlight novel links of an array of factors with sleep health in the aging population and their implications for prevention. Sleep, Circadian Rhythms and Aging Interest Group Sponsored Symposium.

